# Somatotype Variables Related to Muscle Torque and Power in Judoists

**DOI:** 10.2478/v10078-011-0069-y

**Published:** 2011-12-25

**Authors:** Joanna Lewandowska, Krzysztof Buśko, Anna Pastuszak, Katarzyna Boguszewska

**Affiliations:** 1 Department of Anthropology, Josef Pilsudski University of Physical Education, Warsaw, Poland; 2 Department of Biomechanics, Institute of Sport, Warsaw, Poland

**Keywords:** judo, somatotype, strength, power

## Abstract

The purpose of this study was to examine the relationship between somatotype, muscle torque and power output in judoists. Thirteen judoists (age 18.4±3.1 years, body height 178.6±8.2 cm, body mass 82.3±15.9 kg) volunteered to participate in this study. Somatotype was determined using the Heath-Carter method. Maximal muscle torques of elbow, shoulder, knee, hip and trunk flexors as well as extensors were measured under static conditions. Power outputs were measured in 5 maximal cycle ergometer exercise bouts, 10 s each, at increasing external loads equal to 2.5, 5.0, 7.5, 10.0 and 12.5% of body weight. The Pearson’s correlation coefficients were calculated between all parameters. The mean somatotype of judoists was: 3.5-5.9-1.8 (values for endomorphy, mesomorphy and ectomorphy, respectively). The values (mean±SD) of sum of muscle torque of ten muscle groups (TOTAL) was 3702.2±862.9 N x m. The power output ranged from 393.2±79.4 to 1077.2±275.4 W. The values of sum of muscle torque of right and left upper extremities (SUE), sum of muscle torque of right and left lower extremities (SLE), sum of muscle torque of the trunk (ST) and TOTAL were significantly correlated with the mesomorphic component (0.68, 0.80, 0.71 and 0.78, respectively). The ectomorphic component correlated significantly with values of SUE, SLE, ST and TOTAL (−0.69, −0.81, −0.71 and −0.79, respectively). Power output was also strongly correlated with both mesomorphy (positively) and ectomorphy (negatively). The results indicated that the values of mesomorphic and ectomorphic somatotype components influence muscle torque and power output, thus body build could be an important factor affecting results in judo.

## Introduction

In selection of athletes for a particular sport discipline, the focus should be on those traits and abilities which have the most significant influence on sport performance, and on those which are predominantly under the influence of genetic factors. In evaluation of the training process the fitness profile variables enable monitoring of trainable abilities.

The adaptation to physical effort, developed in the course of training and process of selection, resulted in a decrease of somatotype diversity amongst athletes, as compared with non-training population (Tanner, 1964). An even smaller diversity of somatotype can be observed among athletes practicing the same sport and employing the same techniques (Charzewski et al., 1991; Krawczyk et al., 1997; Kuźmicki and Charzewski, 1987). Athletes, representing the highest sport level in disciplines, where body build is one of the most important factors affecting results, exhibit the greatest similarity in morphological traits and motor abilities. Those traits of athletes, achieving highest results in a particular sport, create a somatic and physical “model” for that discipline.

Although judo is a popular contact sport, little research has examined the factors associated with the performance of judoists’ specific skills. Success in judo requires a high level of physical and tactical preparation (Franchini et al., 2005; Thomas et al., 1989). The planning of judo training should not only concern the applied training loads, but it should also focus on the athletes’ physical abilities.

Anthropometric characteristics, maximal strength and power capacities represent essential elements of physical performance in judo. Research into the performance requirements of judoists’ has suggested anthropometric and physical attributes associated with the demands of this sport. Judo competitors should be strongly built, particularly in shoulders and upper parts of the trunk, should have a robust skeleton (large dimensions of knee and elbow breaths) and well developed muscles of legs (especially calf muscles), to enable them to withstand and transmit the forces applied during the fights. As follows from the study of somatotype of athletes representing various sports (Krawczyk et al., 1997) wrestlers and judoists were most robustly built, with the highest level of mesomorphy and very low one of ectomorphy. Research of Claessens et al. (1987) on 38 world top judoists showed that these athletes were mainly mesoendomorphs and indicated that the higher the weight category, the more endomorphic was the somatotype. However, somatotype distribution of world class judoists was very homogeneous. A top judoist is defined by this author, as a broad-developed athlete with high girth values, low content of subcutaneous fat, and “thick-set” stature in relation to body weight. Comparing results of different authors studying the physique of judoists, it can be concluded that the value of mesomorphy increases with increasing sport’s levels of competitors, while the value of endomorphy decreases (Charzewski et al., 1991; Fagerlund and Häkkinen, 1991; Kuźmicki and Charzewski, 1987). Fagerlund and Häkkinen (1991) found significant differences in maximal strength and in the force-velocity relationships between Finnish judoists competing at international, national and recreational levels. Strength profiles of Finnish judoists got worse with a decrease in their sports level, while their body fat content increased.

The measurement of maximal static muscle torque and maximal power output of lower limbs yields valuable information that can be extremely useful in judo training (Häkkinen, 1989). The relationship between strength, power and sport performance of athletes has been documented by several researchers (Carlock et al., 2004; Cronin et al., 2000; Sands et al., 2005; Stone et al., 2002; 2003). Little data are available regarding the relationships between anthropometric characteristics and power, strength and physical fitness of judoists. Quarrie et al. (1996) and Quarrie and Wilson (2000) studied the relationship among anthropometric, strength and power measures of rugby union forwards and their individual force production in the scrum. They found that body mass and somatotype components of the rugby players were significantly associated with their force. Although similar investigations are needed and useful for judo competitors, we found only a few of such in the literature. Farmosi (1980) did not find significant correlations between the characteristics of body build (body weight, body fat, lean body mass, components of somatotype) and motor performance (hand grip and vertical jump) in Hungarian judoists.

The purpose of this study was to examine the relationship between somatotype, muscle torque and power output in judoists.

## Material and Methods

The study was approved by the Local Ethics Committee. All participants were informed about the study aim and methodology, as well as the possibility of immediate resignation at any time of the experiment. Subjects agreed on the above conditions in writing. Thirteen judoists (age 18.4±3.1 years, body height 178.6±8.2 cm, body mass 82.3±15.9 kg, BMI 25.65±3.59, FAT% 10.8±4.0 %) volunteered to participate in the study.

### Maximal muscle torque of ten muscles groups

Flexors and extensors of the elbow, shoulder, hip, knee and trunk were measured in static conditions with the use of a special device (Institute of Sport, Poland) Type SMS1 (upper extremities) and SMS2 (lower extremities and trunk) (Jaszczuk et al., 1987). During the measurement of muscle torque of elbow flexors and extensors the subject was sitting, with his arm bent at a right angle and placed on the armrest, and with the trunk stabilized. The muscle torque of shoulder flexors and extensors was measured in a sitting position. The flexion angle was 70° and the extension angle 50°. The trunk was stabilized and the chest pressed against the testing station. The measurements of muscle torque of knee flexors and extensors were carried out on subjects in a sitting position. The hip and knee joints were bent at 90°. The subjects were stabilized at the level of anterior iliac spines and thighs, with the upper extremities resting on the chest. The subjects were lying face down during the measurement of the muscle torque of hip extensors, and face up during the measurement of the muscle torque of hip flexors. The hip joint angle remained at 90° during measurement. The maximal extension of the elbow, knee and hip joints was accepted as 0°. For the shoulder joint, the positioning of the arm along the side was taken as 0°. The axis of rotation during muscle torque measurement corresponded to the axis of rotation of the torque meter. Muscle torques of the right and left limbs were measured separately, always in the order flexion-extension. Each subject was supposed to achieve the maximal power output during measurement.

### The force-velocity (F–v) and power-velocity (P–v) relationships

Were determined on the basis of results of exercises performed on a Monark 874 E cycloergometer (Sweden) connected to a PC, using the MCE 4.0 software package (“JBA” Zb. Staniak, Poland) (Buśko, 2007). After adjusting the ergometer saddle and handlebars, each subject performed the tests in a stationary position, without lifting off the saddle, with his feet strapped onto the pedals. Each subject performed five 10-second maximal cycloergometer tests with increasing external loads amounting to 2.5, 5.0, 7.5, 10.0 and 12.5% of body weight (BW), respectively. There were 2 min rest periods between the tests. The standard procedures of exercise performance were followed, and the subjects were verbally encouraged to achieve and maintain the maximal pedaling velocity as quickly as possible. With the use of MCE, maximal power output at a given load (*P*_i_; *i* – load value) and velocity (*v*_i_) necessary to achieve *P*_i_ were determined (Buśko, 2007).

### Anthropometry

Included the following variables: body height, body mass, four skinfolds (over triceps, subscapular, suprailiac, medial-calf), biceps girth (flexed 90° and tensed), standing calf girth, bicondylar femur breadth, bicondylar humerus breadth (Norton et al., 1996). Body height was determined using a SiberHegner anthropometer (Switzerland), body mass was evaluated on an electronic scale (Tanita TBF 300, Japan), skinfolds were measured using a Harpenden skinfold caliper, girth measurements were acquired by using a steel measuring tape and bicondylar diameters of femur and humerus were measured using small spreading caliper (SiberHegner, Switzerland).

All measurements were taken in duplicate and performed by the same investigator applying standard anthropometrical methods according to the procedure of the International Biological Programme (Weiner and Lourie, 1969). The measurements were performed before the second preparatory period.

### Somatotypes

Were calculated by the Heath-Carter method (Carter, 1996).

Relationships between muscle torques, power output and components of somatotype were assessed by calculating the Pearson’s linear correlation coefficients. The level of statistical significance was set at p<0.05. All statistical calculations were conducted using a Statistica^™^ program (v. 8.0, StatSoft 2007)

## Results

**Figure 1:**
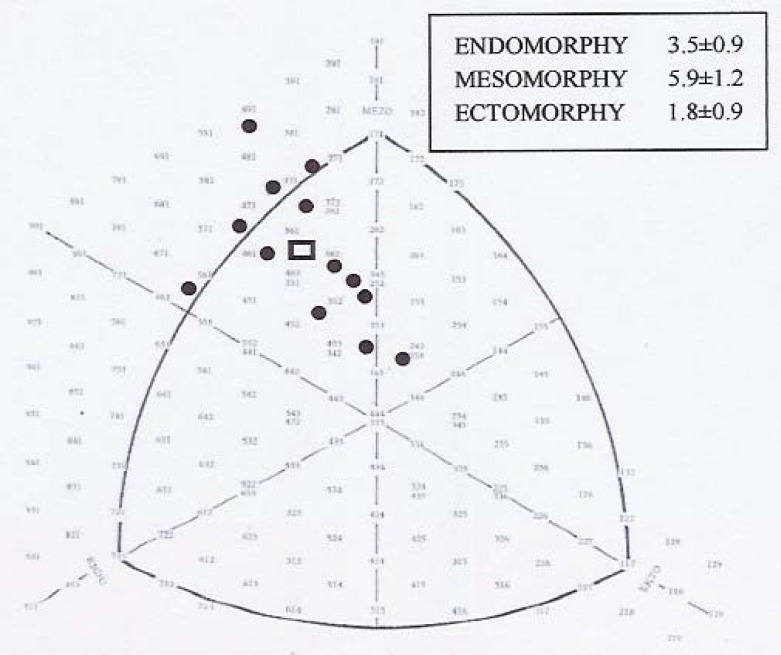
Somatochart of judoists, n = 13. The circles are the individual somatotypes, the square is the mean somatotype.

The mean somatotype of judoists was: 3.5-5.9-1.8 (values for endomorphy, mesomorphy and ectomorphy, respectively; Figure 1.) The results of the strength assessments of the judo athletes and the association between muscle torques and components of somatotype are presented in Table 1. The values (mean±SD) of sum of muscle torque of ten muscle groups (TOTAL) was 3702.2±862.9 N x m. The values of sum of muscle torque of right and left upper extremities (SUE), sum of muscle torque of right and left lower extremities (SLE), sum of muscle torque of the trunk (ST) and TOTAL were significantly correlated with mesomorphic component: r = 0.68, 0.80, 0.71 and 0.78, respectively. Ectomorphic component correlated significantly with the values of SUE, SLE, ST and TOTAL: r = −0.69, −0.81, −0.71 and −0.79, respectively. The correlations between endomorphy and the sums of muscle torque were not significant.

The power output ranged from 393.2±79.4 to 1077.2±275.4 W (Table 2). Power output was strongly correlated with both mesomorphy (positively) and ectomorphy (negatively). No significant correlation between power output and endomorphy was found.

## Discussion

In this study judo athletes had a similar mean somatotype profile (3.5-5.9-1.8) as elite judoists (2.7–6.3–1.7) in Kuźmicki and Charzewski’s study (1987), but they had a lower level of mesomorphy and higher value of endomorphy. The mean somatotype of judoists in our study was very similar to the average somatotype (3.61-6.97-1.56) of the 18 Hungarian judoists selected for the national team examined by Farmosi (1980), however, the value of mesomorphy was also higher in the Hungarian judoists than in our study. Although, there were differences in the values of various components between compared groups, the mesomorphic factor clearly dominated in both of them. In quoted by Kuźmicki and Charzewski (1987) Carter’s study, mean somatotype of judo athletes participating in the Olympic Games in Montreal was 2.0-6.5-1.3. Those results indicate massive (mesomorphic) body build of judoists, characterized by large circumferences of muscles and developed (robust) bone bases, adapted to heavy loads. The diversity of somatotypes observed in elite judoists (Krawczyk et al., 1997; Kuźmicki and Charzewski, 1987) was lower than in the studied group of young judoists. This is in accordance with the reports of Charzewski et al. (1991), Krawczyk et al. (1997) and Kuźmicki and Charzewski (1987), who observed that top level athletes, achieving the highest results in a particular sport discipline, exhibited the greatest similarity in somatic build and form the most homogenous morphological group. Somatotype as well as motor performance of athletes depends on their sport level. The mean values of muscle torques among judoists of national caliber in this study were lower than muscle torques of judoists who had been selected to Polish national team and examined by Buśko and Nowak (2008). Both the muscle torques as well as power output developed by judoists in our study were higher than in the study conducted on junior judoists by Boguszewska et al. (2010).

Research of body build of judoists as well as their motor performance has suggested the existence of associations between them, however, these relationships were examined only in a few studies (Farmosi, 1980). Quarrie and Wilson (2000) studied the relationship among anthropometric, strength and power measures of rugby union forwards and their individual force production in the scrum. The body mass and somatotype of the players were significantly associated with the force they applied in the scrum. Heavier athletes and those who were more endo-mesomorphic, were capable of producing higher individual scrummaging forces than those who were lighter or more ectomorphic. All three components of somatotype (endomorphy, mesomorphy, ectomorphy) correlated significantly with scrum force of rugby players. In our study, like in the Quarrie and Wilson’s (2000) paper, the somatotype components in examined judoists were significantly associated with strength and power output. The strength and power assessments of the judo athletes were highly correlated with mesomorphy (positively) and ectomorphy (negatively). The torque and power assessment variables did not correlate with endomorphy. Although the correlation between endomorphy and strength and power was not significant, weak positive association between these variables was found. This is in agreement with results of other authors, who found positive correlation between body mass or/and endomorphy and sport results in athletes of sports demanding meso-endomorphic body build (Janiak at al., 1993; Katič, 1996; Quarrie and Wilson, 2000). Janiak et al. (1993) found positive correlations between muscle torques or their sum and body mass in rowers. Siahkouhian and Hedayatneja (2010) showed that the snatch and clean & jerk records significantly correlated with body height, sitting height, body weight, shoulder and chest circumference, LBM, BMI. Katič’s (1996) results indicated that a well developed musculature, large transversal skeletal dimensions and a moderate contribution of endomorphy, is favorable to throwing. Body fat is a component of total body mass, what could explain a positive correlation between endomorphy and strength and power output. Such results obtained in judoists were connected with their specific body build, which characterized moderate level of endomorphy as a result of high fat content compared with other sports. Krawczyk et al. (1995) studied elite athletes from diverse sports, with respect to their body components (lean body mass and fat content). They found that judoists belonged to a group of athletes, who had higher total body fat (10.28% ± 2.15% in men), and very high variability in relative body fat content. In the study of Fagerlund and Häkkinen (1991), the percentage of body fat in Finnish judoists at the recreational, national and international level was higher than in studies quoted above, but body fat decreased clearly with increasing sports level and amounted to 15.9%, 14.6% and 13.6%, respectively.

On the other hand, investigations of elite Polish judoists conducted by Kuźmicki and Charzewski (1987), Charzewski and Kuźmicki (1989) showed that within the period of three years of training, somatotypes of competitors who achieved better results, changed displaying the increase of mesomorphy and a decrease of endoand ectomorphy. The contribution of mesomorphy in somatotype represents lean body mass (LBM) level and the value of endomorphy reflects body fatness. Body composition was found to play a significant role in anaerobic power, aerobic capacity and sport results of athletes. Anaerobic power in Olympic weight lifters showed a significant positive correlation with LBM and a negative one with body fat (Pilis et al., 1997). In our study, mesomorphy significantly correlated with anaerobic power.

## Conclusion

The results of this study indicated that a higher mesomorphy and a lower ectomorphy were important factors determining strength and power output in judoists.

Muscle torques and power output developed on a cycle ergometer showed significant positive correlations with the mesomorphic component while significant negative ones with ectomorphy.

## Figures and Tables

**Table 1:** Mean values (±SD) of the sums of the maximal muscle torque of the right (R) and left (L) upper extremity (SUE), lower extremity (SLE), trunk (ST) and all ten muscle groups (TOTAL), and the Pearson’s linear correlation coefficients between muscle torque and endomorphy (ENDO), mesomorphy (MESO) and ectomorphy (ECTO)

Variables	Muscle torque [N·m] (Mean ± SD)	ENDO (r)	MESO (r)	ECTO (r)
SUER	298.3±75.3	0.12	0.61*	−0.63*
SUEL	294.6±67.4	0.15	0.73*	−0.75*
SLER	1131.5±245.7	0.05	0.73*	−0.74*
SLEL	1127.1±280.4	0.08	0.85*	−0.85*
ST	850.6±223.5	0.17	0.71*	−0.71*
TOTAL	3702.2±862.9	0.11	0.78*	−0.79*

* - p<0.05

**Table 2:** Mean values (±SD) of power output (P) and the Pearson’s linear correlation coefficients between power output and endomorphy (ENDO), mesomorphy (MESO) and ectomorphy (ECTO)

Load [% BW]	P [W] (Mean ± SD)	ENDO (r)	MESO (r)	ECTO (r)
2.5% BW	393.2±79.4	0.24	0.74*	−0.80*
5.0% BW	724.3±151.6	0.17	0.73*	−0.79*
7.5% BW	935.7±215.9	0.21	0.65*	−0.72*
10.0% BW	1038.5±240.3	0.15	0.55*	−0.61*
12.5% BW	1077.2±275.4	−0.02	0.62*	−0.60*

* - p<0.05
